# **Use of Platelet Rich Plasma (PRP) and Hyaluronic Acid in Treatment of Extremity Gunshot Injuries: A Case Report**

**Published:** 2016-01

**Authors:** Valerio Cervelli, Pietro Gentile, Lorenzo Brinci, Camilla Di Pasquali, Ilaria Bocchini, Barbara De Angelis

**Affiliations:** 1 Department of Plastic and Reconstructive Surgery, University of Rome, Tor Vergata, Italy;; 2 Department of Regenerative Surgery, University of Rome, Tor Vergata, Rome, Italy

**Keywords:** Platelet rich plasma (PRP), Hyaluronic acid (HA), Extremity, Gunshot injuries

**DEAR EDITOR**

In the view of efficiency and cost effectiveness the increasing incidence of gunshot wounds demands a modification of treatment protocols. The general basis are antibiotics, fracture stabilization and debridement of soft tissue injuries. The pathology of gunshot wounds and established treatment algorithm however have to be adjusted to the ongoing development of gun technology and the thereby caused specific lesions. In most of the cases it is difficult to obtain a complete healing, because of the complexity of different tissues. Moreover the presence of foreign bodies and the tendency to develop infections, slow down the healing process.^1^

There are many ways to treat firearm wound such as advanced dressings, negative pressure wound therapy, surgical toilet, dermal substitute and autologous skin grafting, free or local flaps. It depends on the extent of lesion, the type of firearm, the localization and the general health of patient.^2^ The purpose of our case report was to determine how platelet rich plasma (PRP) and hyaluronic acid (HA) help healing of extremity gunshot injuries.

A 52-year-old male admitted to our department presented with a gunshot wound of lower left limb without comorbidities, smoker of 50 cigarettes per day. The patient was accidentally insult by a shotgun in 9 December 2008. He was instantly brought to the hospital emergency room. In the emergency room, the initial assessment entire patient was performed by the trauma team, then the wound was sterilely dressed and a splint was applied to the limb. This wound was characterized by the presence of the shotgun cartridge wad and widespread contamination from foreign materials shredded in the wound by the shot blast. The initial depth of the wound was 4 cm and width was 3.6 cm (Figure 1).

**Fig. 1:**
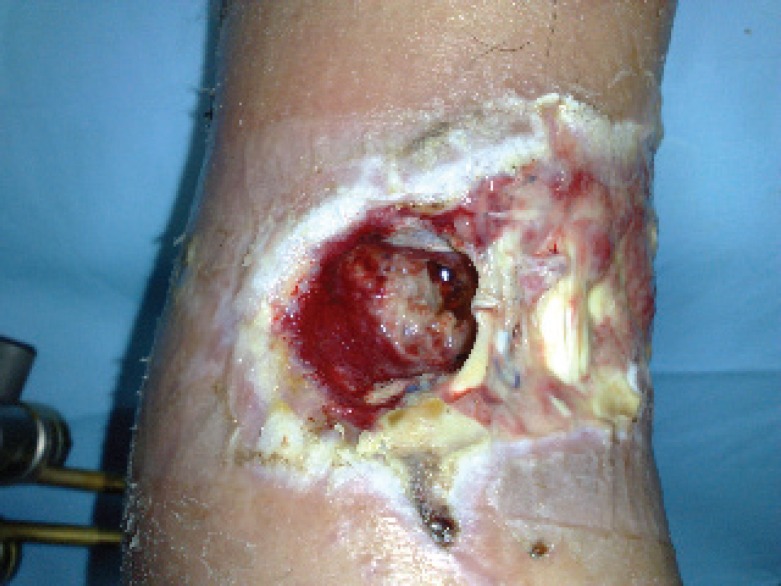
Post-traumatic wound before the treatment

In the meantime the patient was underwent radiographic evaluation. The purpose of a radiographic examination was not only to assess the extent of hard and soft tissue destruction but also locate foreign bodies and plan surgery. Conventional radiography does depict the bullet and its site, subcutaneous emphysema, blow-out fractures and the location of bone splinters. This permits adequate emergency surgery and an efficacious orthopedic approach, as well as selection of the cases to be submitted to clinical monitoring. The radiographic examination showed a multi-fragmented fracture of distal end of tibia and also a fracture of fibula, well as numerous radiopaque foreign bodies (Figure 2). 

**Fig. 2:**
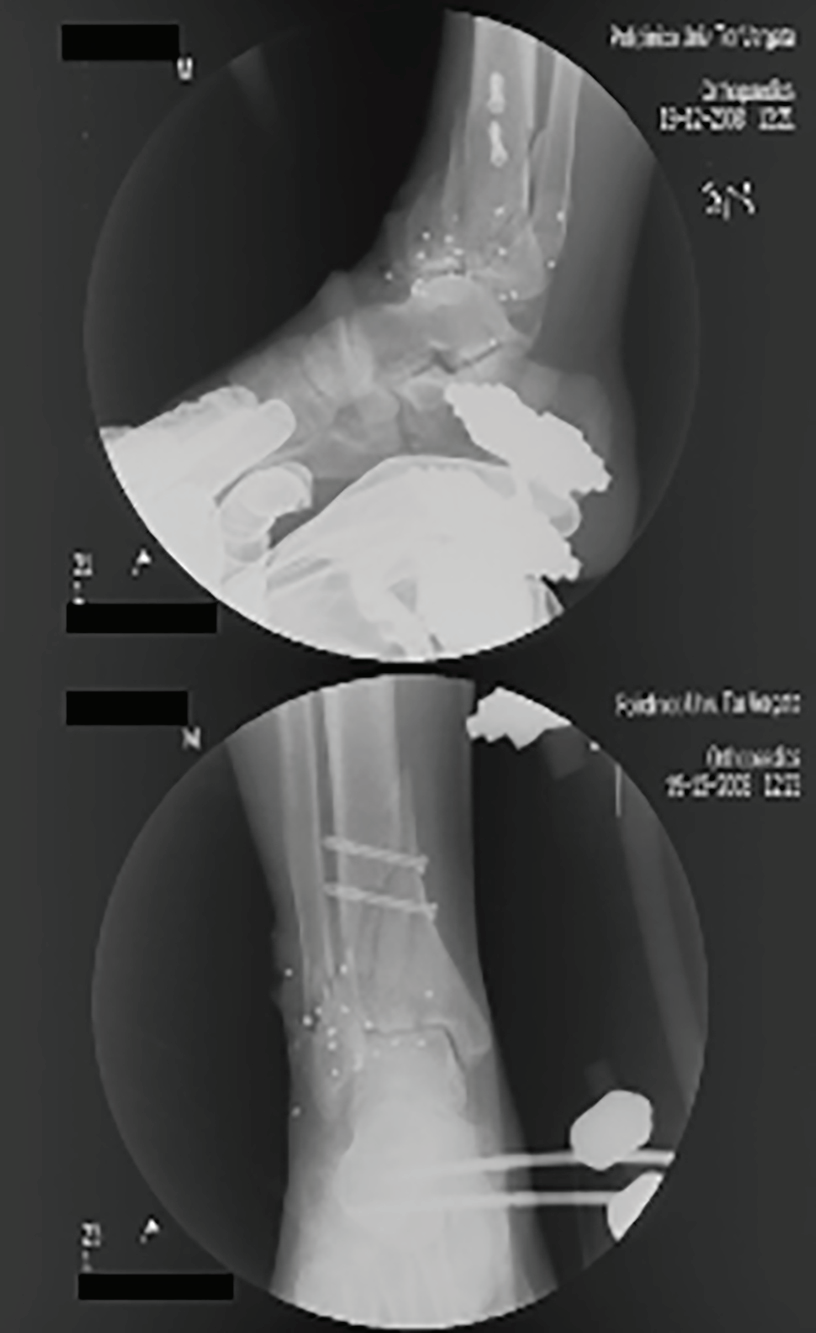
Radiographic examination: multi-fragmented fracture of distal end of tibia and also a fracture of fibula, well as numerous radiopaque foreign bodies

After the response from the radiograph, the patient was subjected to an operation in which was performed an adequate debridement of wound to remove unhealthy tissue and foreign bodies, but it was not possible to completely remove all fragments. Failure to adhere to the basic principle will place the patient at an increased risk for infection and its sequelae. Afterwards two internal screws were placed and an external fixator was placed away from the site of injury to stabilize the wound and the joints in an effort to minimize soft-tissue compromise. After stabilization of the limb, the patient’s wound haven’t healed and it became a traumatic ulcer.

Temporary coverage of exposed tissue was obtained by placement of sterile dressing and then the patient was invited to Department of Plastic and Reconstructive Surgery, University of Rome, Tor Vergata, Italy in collaboration with the Transfusion Center of Policlinico Casilino, Rome. After careful evaluation that showed a PDS of lower left limb with flexor tendon exposure and a cavity with bone exposure, and also with multiple foreign bodies, we decided to actuate a therapeutic plane. The patient was treated initially for 20 days biweekly with advanced dressing consisted on the use of a Polyurethane sponge with Hydrogel. After this period he was undergone to PRP and HA procedure in peripheral anesthesia.

A self-contained disposable kit was used to process 8-10 ml of venous peripheral blood. The kit consists of two or more sterile evacuated blood collection tubes, needles, and a transfer device. Blood was collected in one tube (8ml each) and autologous thrombin was obtained treating 8 ml patient’s blood using another tube. All tubes were centrifuged at 1500 g (corresponding to 3000 rpm) and PRP was obtained. PRP obtained is a high quality PRP with the highest platelet recovery and highest growth factors contents as described before.^3^^,^^4^ PRP activated with autologous thrombin may be injected intra-lesional and perilesional or forming platelet gel and used topically (Figure 3). In combination with the platelet gel, the wound was covered with a three-dimensional polymerized hyaluronic acid medicated biologic dressing (Figure 4). An elasto-compression has been performed to allow a better flow at venous-lymphatic level of the leg in order to interfere as little as possible with the regeneration process. During the surgery some foreign bodies were removed, while some foreign bodies were located in deep. The patient was treated with prophylactic antibiotic twice daily for 1 week starting 1 day prior to the surgical treatment.

**Fig. 3:**
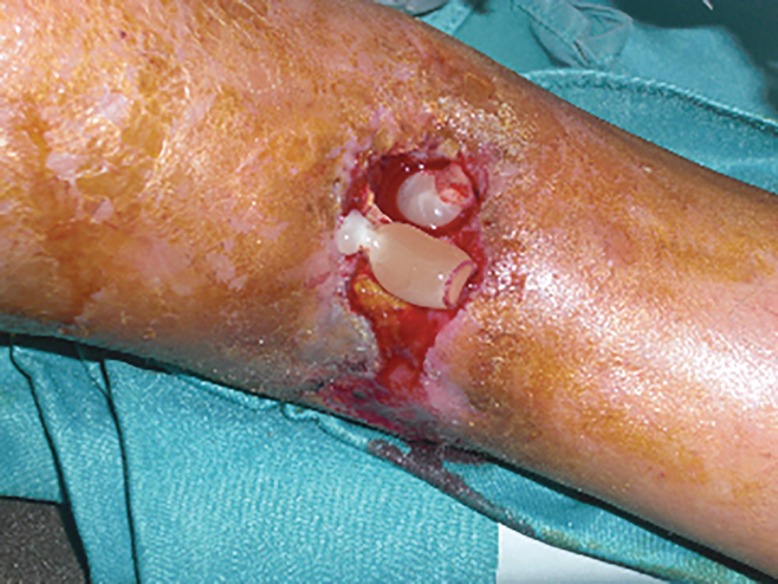
Intra-operatory view. PRP gel application

**Fig. 4:**
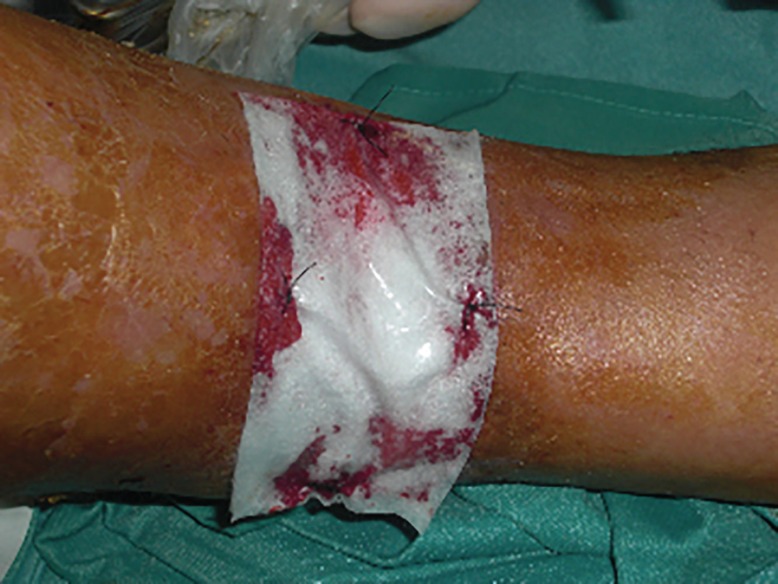
Intra-operatory view. Application of HA on PRP

Postoperative follow-up consisted of four visits during two months– one for each week– and two additional visits at the third and sixth month. Fifteen days later we have removed silicon’s film and after three weeks we removed the excess of HA that wasn’t integrated with the wound. Thirty days after the surgical treatment we applied a HA. Forty-five days after the surgical treatment there was full recovery (Figure 5). After that the patient applied emollient, moisturizing and firming creams and a knee-high elastic compression.

**Fig. 5:**
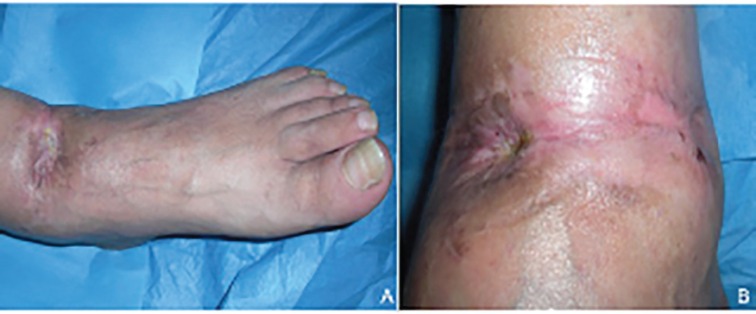
Postoperative view after 45 days: A: anterior view, B: antero-medial view

With the help of a physical therapist the patient’s active and passive range of motion in a single plane was evaluated at the first and the sixth months from surgery. Secondary endpoints were the assessment of infections, inflammations or any adverse effects of the PRP and HA procedure, particular medications assumed, postoperative pain (evaluated with the visual-analogic scale-VAS). The postoperative course was uneventful and the early postoperative result was very satisfying for both the surgeon and the patient (Figure 6). The use of PRP and HA in our patient was beneficial for promotion of healing in the area surrounding the wound, with an increase of blood supply, a restored trophism, pain reduction and the decline of wound secretion. The wound infection had been tested with negative tampon confirmation performed at 2 weeks interval for 4 times. 

**Fig. 6:**
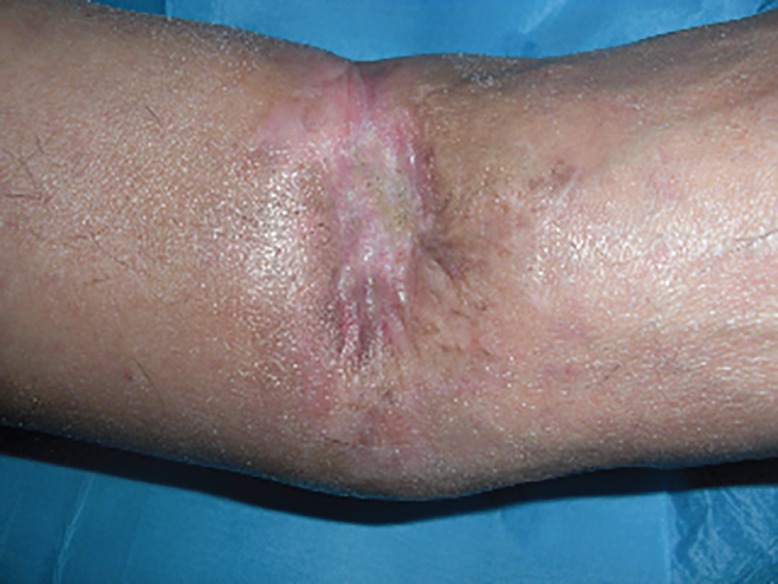
Postoperative view after 8 weeks

Wound healing due to gunshot injuries is an evolutionarily conserved complex multicellular process that in skin aims at barrier restoration. The process involves the coordinated efforts of several cell types including keratinocytes, fibroblasts, endothelial cells, macrophages and platelets. The migration, infiltration, proliferation and differentiation of these cells will culminate in an inflammatory response, the formation of new tissue and ultimately wound closure. This complex process is executed and regulated by an equally complex signalling network involving numerous growth factors, cytokines and chemokines. Particular relevance is the epidermal growth factor (EGF) family, transforming growth factor beta (TGF-beta) family, fibroblast growth factor (FGF) family, vascular endothelial growth factor (VEGF), granulocyte macrophage colony stimulating factor (GM-CSF), platelet-derived growth factor (PDGF), connective tissue growth factor (CTGF), interleukin (IL) family, and tumour necrosis factor-alpha family.^5^

Gunshot injuries around the foot are often associated with significant skin loss, which results in the exposure of tendons, bone, or hardware, and associated wound-management difficulties. The rapid formation of granulation tissue and blood vessels are essential for the healing of these wounds. Traditionally frequent wet dressing changes (3–4 times/day) are used to treat such cases, but this treatment is protracted and painful. Furthermore, interstitial fluid from open wounds reduces local blood supply and disturbs wound healing due to its collagenase and metalloproteinase constituents. Soft tissue defects in the foot or leg region usually require local or free flap surgery when a skin graft procedure is not applicable due to limited granulation tissue formation.^1^ A split-thickness skin graft is not recommended for wounds with exposed bone or neurovascular structures.

An ideal scaffold should promote rapid remodelling, possess increased strength, demonstrate improved healing, and permit early rehabilitation and return to function after implantation. In addition, the availability of such a scaffold would eliminate the donor site morbidity and infection too.^6^ In the present study, the severities of open gunshot wound was noticeably reduced after treatment with scaffolds of PRP and HA together even this study had several limitations, namely; the small data size, the absence of a control group, which reduced objectivity. Based on the results of previous studies it appears that PRP and HA together play a significant role in the formation of granulation tissue and in the prevention of infection.^7^^,^^8^

Our results add to growing evidence that PRP and HA is a useful adjunctive treatment for open gunshot wounds. In the present study, it was found to facilitate the rapid formation of granulation tissue, to shorten healing time, and to reduce remarkably the need for additional soft tissue reconstructive surgery. The minimally invasive technique is well accepted by patients with a noteworthy improvement of quality of life along with cost reduction due to the fewer number of medications. The best treatment strategy can be the integration of different but complementary disciplines such as cell therapy, bio-engineering and biomaterials sciences as effective support to the surgical procedure. So our findings demonstrated the ability of the combination of PRP and HA to regenerate tissue and epithelization with wound closure, with a significant healing time reduction. Furthermore as the minimally invasive technique is well accepted by the patient with a noteworthy improvement of the quality of life along with cost reduction due to the fewer number of medications, it can be recommended as a safe therapy.

## CONFLICT OF INTEREST

None of the Authors has a financial interest in any products and in any other devices or drugs mentioned in this article. No funding was received for the research reported in the article.
